# Occult Breast Carcinoma Presenting as Gastrointestinal Metastases

**DOI:** 10.1155/2009/564756

**Published:** 2009-12-22

**Authors:** Lonzetta Neal, Nicole Sookhan, Carol Reynolds

**Affiliations:** Divisions of General Internal Medicine Breast Diagnostic Clinic, and Pathology, Department of Surgery, Mayo Clinic College of Medicine, 200 First Street SW, Rochester, MN 55905, USA

## Abstract

Occult breast cancer has frequently been described as presenting as axillary lymph node metastases but rarely as gastrointestinal metastases, Varadarajan et al. (2007). In extremely rare situations, cancerous lesions identified in the gastrointestinal tract have been determined to be metastatic lesions from primary breast cancers, Taal et al. (2000). We report a case of an occult lobular adenocarcinoma presenting as gastrointestinal metastases. It is essential that the possibility of lesions found in the gastrointestinal tract originating from distant or occult cancers be considered in order that appropriate therapeutic options may be discussed and considered early after diagnosis.

## 1. Introduction

Breast carcinoma is the most common malignancy in women after skin cancer and, after lung cancer, the most frequent cause of cancer death in females. Over 175,000 women were diagnosed with breast cancer in 2007 and more than 40,000 died from the disease. With advancements in screening over the past 25 years, fewer than ten percent of patients will present with metastatic breast carcinoma at the time of diagnosis. However, despite improvements in surgical and chemotherapeutic therapies, women with early-stage and locally advanced breast cancer relapse not infrequently, usually presenting with distant metastatic disease. Metastatic disease of breast cancer origin commonly appears in the skeleton (43%), stomach (27%), lung (8%), and liver (4%) [1]. Occult breast cancer (OBC) can rarely manifest as axillary nodal metastases with an incidence of 0.3% to 1% [2], and, of those female patients presenting with adenocarcinoma within an axillary lymph node, breast cancer is most likely the primary source [3]. Gastrointestinal (GI) tract metastasis and carcinomatosis from primary breast cancer are rare. Data from two series, including surgical and autopsy specimens targeting the gastrointestinal tract, reported metastatic breast cancer occurred in this site at a rate of 8% to 35% [4, 5].

Our review of the literature revealed one case report of occult breast carcinoma presenting as a pancreatic tumor [6]. We report a case of an occult breast cancer presenting with gastrointestinal metastasis.

## 2. Background

### 2.1. Case Report

A 53-year-old female presented to an outside institution with a one-month complaint of abdominal pain. An extensive evaluation was only remarkable for an umbilical hernia and a chest wall nodule. The chest wall nodule was excised and reported as benign. Screening colonoscopy was negative except for a reportedly benign colon polyp that was removed. She underwent an umbilical hernia repair with mesh placement. Her postoperative course was complicated by omental herniation under the mesh with omental infarction and intraperitoneal bleeding. She underwent repair of this process but continued to feel anorexic and ill. Five months following her initial operation, she developed borborygmi associated with watery diarrhea occurring up to ten times a day. 

An upper gastrointestinal endoscopic study with biopsies of the antrum was performed. No evidence of *H. pylori* or celiac sprue was noted. The biopsy revealed metastatic carcinoma consistent with a breast primary. Estrogen receptor was strongly positive and progesterone receptor was negative. HER2 was negative by FISH. She offered no breast complaints at the time of diagnosis. Clinical examination of the breasts and axillae was normal. Screening mammograms performed previously showed heterogeneously dense nodular parenchyma bilaterally. Minimal architectural distortion was present in the superior portion of the left breast but this was previously noted and felt to be benign. Family history was remarkable for a maternal aunt and a paternal aunt with postmenopausal breast carcinoma. 

Given her unusual pathology, all of her prior pathology was sent to our institution for review. The previously resected colon polyp and chest wall lesion were reevaluated and found to be consistent with metastatic breast carcinoma. She underwent a bilateral breast MRI examination, which showed no worrisome areas of focal enhancement. She was then started on with hormonal therapy with an aromatase inhibitor.

## 3. Pathological Findings

The stomach biopsy specimen showed metastatic carcinoma consistent with breast primary. Immunohistochemical stains CK7, CK20, CD45, CDX2, estrogen receptor (ER), progesterone receptor (PR), and HER2 were performed. The neoplastic cells were ER positive (>95% nuclear staining), PR weakly positive (10% nuclear staining), and CK7 positive. Tumor stainings for HER2, CK20, CD45, and CDX2 were negative. The pattern of tumor infiltration, morphology and immunohistochemical profiles were consistent with lobular breast carcinoma. The stomach lesion exhibited a linitus plastica appearance.

The chest wall lesion was also consistent with metastatic breast carcinoma which was ER positive, PR weakly positive, and equivocal for HER2 (score 2+) with negative HER2 gene amplification by FISH. 

## 4. Discussion

Occult breast carcinoma may rarely present as axillary metastasis [2]; however, presentation as gastrointestinal metastasis has never been described. Metastases from gastrointestinal system have been reported after a previous diagnosis of breast carcinoma within a period of months to years between the original diagnosis of breast cancer and recurrence in the gastrointestinal tract [1, 7]. Symptoms of gastrointestinal metastases can be very nonspecific in their presentation. In this case report, the gastrointestinal metastasis was an incidental finding, and retrospective review of histology revealed that this was of breast origin.

The metastatic patterns of lobular and ductal carcinoma have been reported to differ considerably. Metastatic breast carcinomas of ductal origin usually present with hepatic, lung, brain, and bone metastases whereas metastatic lobular carcinoma is noted to spread to gastrointestinal, gynecological, and peritoneal structures [8]. Autopsy series report the distribution of gastrointestinal metastases as follows: esophagus (25%), stomach (25%), small intestine (28%), colon (19%), and rectum (4%) [5].

 Cormier et al. [9] reported six patients with linitis plastica attributable to metastatic breast cancer. All six cases were associated with lobular breast carcinoma. A 30-year review by the same group revealed 25 additional cases in which lobular carcinoma and linitis plastica due to metastasis occurred metachronously or synchronously in patients. Most of the women (74%) had metastasis within 5 years of the initial diagnosis of breast cancer; however, four patients presented with metastatic gastric disease more than 10 years after the identification of the primary breast cancer [9]. McLemore et al. [10] found that 73 patients out of 12,001 with metastatic disease secondary to breast cancer had GI tract involvement. The mean interval between primary breast cancers to GI presentation was 7 years. However, 16% of patients were found to have peritoneal metastatic disease at the time of their breast cancer diagnosis.

Abdominal pain was found to be the most common symptom of breast cancer metastatic to the gastrointestinal tract, followed by bloating, melena, GI hemorrhage, bowel obstruction, early satiety, dysphagia, weight loss, anemia, fatigue, and a palpable abdominal mass [10].

Metastatic breast cancer can easily be mistaken for a primary gastrointestinal cancer [11, 12]. Schwartz et al. [11] found that seven patients who presented with gastrointestinal symptoms were initially thought to have primary GI cancer, one with a nearly obstructing colon tumor. These patients were found to have primary breast cancer after subsequent comparisons with prior breast specimens.

Metastatic breast cancer can be difficult to distinguish from primary gastric cancer. Metastasis to the stomach from lobular type carcinoma tends to exhibit tumor cells that are infiltrating in single file process between benign gastric glands. Signet cells may be occasionally noted. This histologic finding resembles primary diffuse type gastric carcinomas (linitis plastica). Metastasis from ductal type carcinoma can resemble poorly differentiated intestinal type gastric adenocarcinomas [11]. Immunohistochemical markers for estrogen receptor and differential cytokeratin expression (cytokeratin 7 and cytokeratin 20) can allow accurate diagnosis. It is important to make the distinction between a primary or a metastatic breast carcinoma. The administration of chemotherapy and hormonal therapy can significantly improve the survival in patients with metastatic breast carcinoma [9, 10]. There is a palliative role for surgery for the relief of symptoms such as pain from mass effect, obstruction, or dysphagia. In general, gastric metastases from breast cancer reflect a poor prognosis despite chemotherapy and hormonal therapy. Median survival time from the time of diagnosis of metastases was noted to be approximately 28 months [10].

## 5. Conclusion

It is important for physicians who follow survivors of invasive lobular carcinoma of the breast to remember that gastrointestinal symptoms can be a manifestation of metastatic disease. The disease free interval between primary breast cancer and gastrointestinal involvement may be as long as 10 years from the time of diagnosis. In our unique case, the breast primary was not found. Once the correct diagnosis is made, targeted systemic treatment specific for breast cancer may be considered and initiated if appropriate.

## Figures and Tables

**Figure 1:**
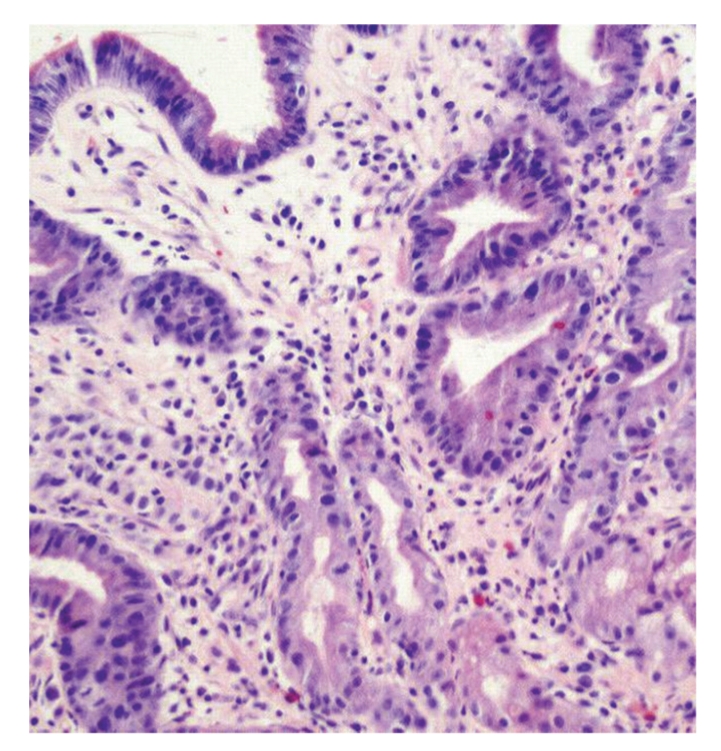
Gastric biopsy. Infiltrating tumor cells (arrow) involving gastric antrum wall (hematoxylin and eosin). Original magnification ×200.

**Figure 2:**
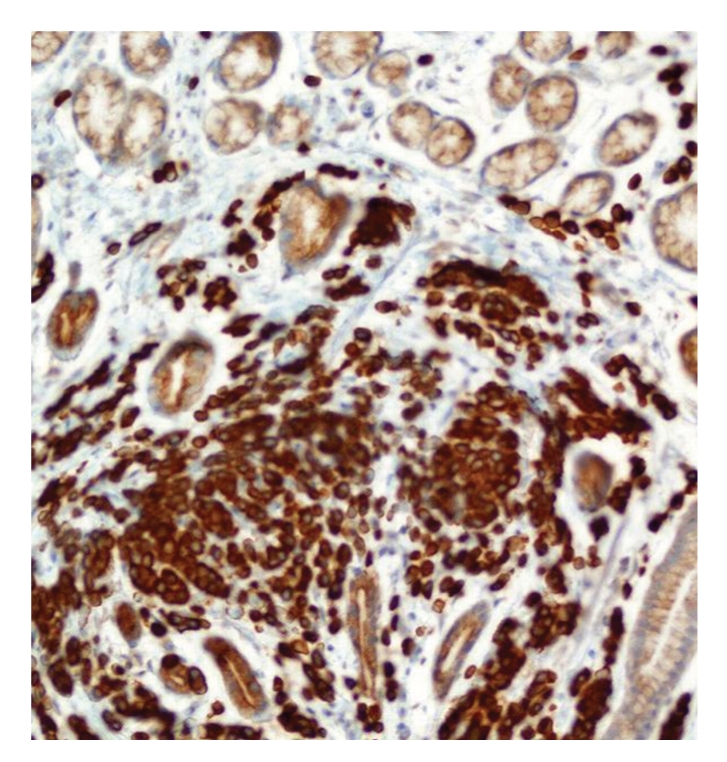
Gastric biopsy. Numerous cytokeratin 7, CK7-positive tumor cells infiltrating gastric wall. Original magnification ×200.

**Figure 3:**
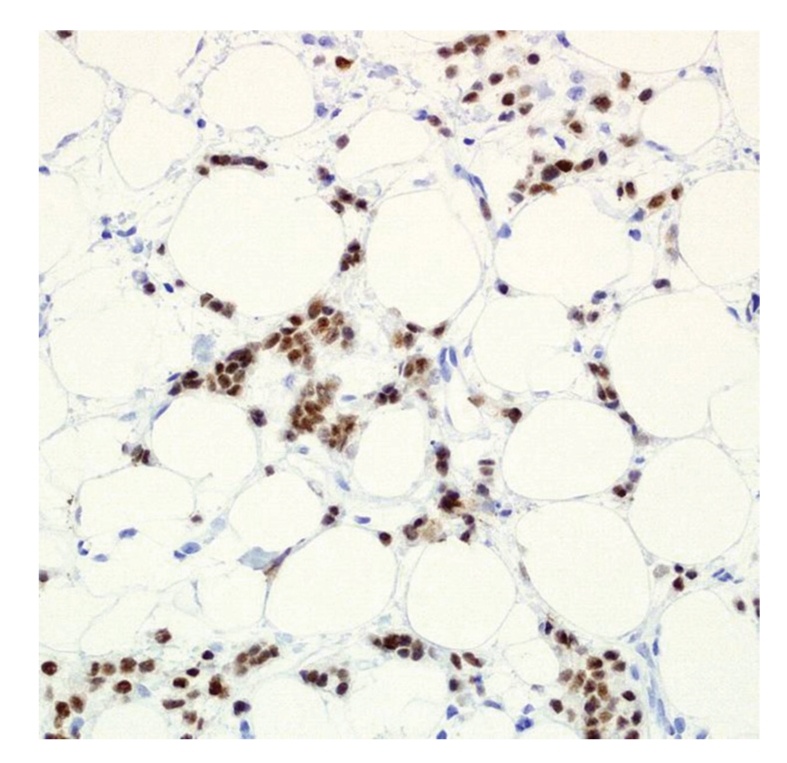
Chest wall lesion. Infiltrating estrogen receptor, ER-positive tumor cells amongst fibrous septa consistent with metastatic breast carcinoma. Original magnification ×100.
